# The steady state visual evoked potential (SSVEP) tracks “sticky” thinking, but not more general mind-wandering

**DOI:** 10.3389/fnhum.2022.892863

**Published:** 2022-08-11

**Authors:** Hang Yang, Ken A. Paller, Marieke van Vugt

**Affiliations:** ^1^Bernoulli Institute for Mathematics, Computer Science and Artificial Intelligence, University of Groningen, Groningen, Netherlands; ^2^Department of Psychology, Northwestern University, Evanston, IL, United States

**Keywords:** spontaneous thought, mind-wandering, SSVEP, machine learning, MVPA

## Abstract

For a large proportion of our daily lives, spontaneously occurring thoughts tend to disengage our minds from goal-directed thinking. Previous studies showed that EEG features such as the P3 and alpha oscillations can predict mind-wandering to some extent, but only with accuracies of around 60%. A potential candidate for improving prediction accuracy is the Steady-State Visual Evoked Potential (SSVEP), which is used frequently in single-trial contexts such as brain-computer interfaces as a marker of the direction of attention. In this study, we modified the sustained attention to response task (SART) that is usually employed to measure spontaneous thought to incorporate the SSVEP elicited by a 12.5-Hz flicker. We then examined whether the SSVEP could track and allow for the prediction of the stickiness and task-relatedness dimensions of spontaneous thought. Our results show that the SSVEP evoked by flickering words was able to distinguish between more and less sticky thinking but not between whether a participant was on- or off-task. This suggests that the SSVEP is able to track spontaneous thinking when it is strongly disengaged from the task (as in the sticky form of off-task thinking) but not off-task thought in general. Future research should determine the exact dimensions of spontaneous thought to which the SSVEP is most sensitive.

## Introduction

Frequently, when we try to engage with a specific task such as reading an article, our thoughts may spontaneously shift to other matters (Killingsworth and Gilbert, 2010). These thoughts can remove us from the current space and time by means of our imagination (McVay and Kane, 2012a). When spontaneous thoughts arise during driving, for example, our attention may shift from the act of driving to contemplating what to have for dinner (Smallwood and Schooler, 2015). Such spontaneous thoughts can be inconsequential, but when unexpected things happen on the road the results can be tragic. Therefore, it would be of great benefit if spontaneous thoughts could be tracked in real time. One method for tracking spontaneous thoughts makes use of electroencephalography (EEG), which has a very high temporal resolution.

**Figure 1:**
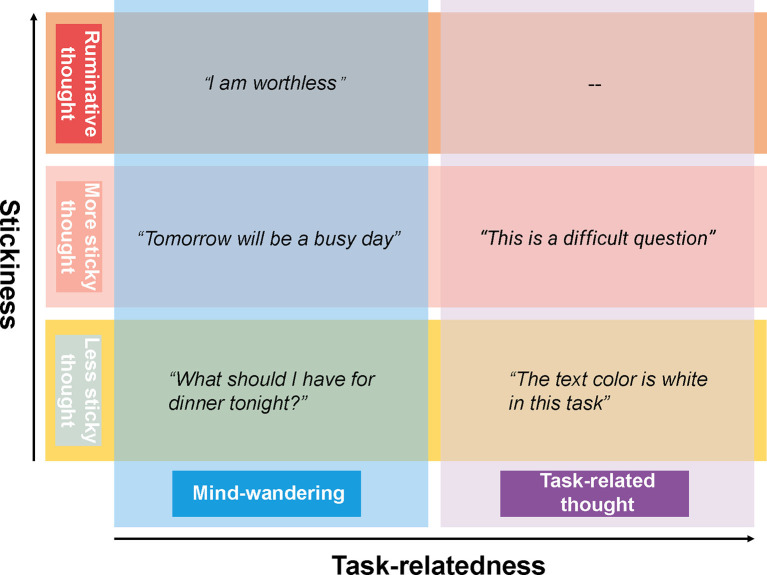
The framework of spontaneous thoughts and the examples, the horizontal axis shows the task-relatedness dimension of spontaneous thoughts while the vertical axis shows the stickiness dimension.

Spontaneous thought has been defined in recent decades as a sequence of mental states, that arise relatively freely due to an absence of constraints on the contents of each state and on the transitions from one mental state to another (Christoff et al., 2016). Within this framework, there are two dimensions of spontaneous thoughts that are commonly studied. The first dimension is the task-relatedness, i.e., when we deliberately shift our attention to a task-unrelated thought, mind-wandering occurs (Figure 1). Mind-wandering has been defined as self-generated thought, stimulus-independent thought, or task-unrelated thought in the scientific literature (Smallwood and Schooler, 2015). The second dimension of spontaneous thought is one that is gaining more and more interest and has been referred to by Christoff as the level of constraints on thought. The level of constraints can be measured in various ways, including by asking participants to rate the degree to which thoughts are immersive or difficult to disengage from, i.e., the subjective sense of “stickiness” of the thoughts. For instance, poor performance in a job interview might trigger a flow of thoughts which are difficult to let go of such as “I am a failure, I am worthless.” We think of such “sticky thinking” as a pre-clinical manifestation of ruminative thinking—repetitive thoughts constrained on a single topic or theme that are often negative and self-related. This mode of thinking is common to clinical syndromes such as depression (Christoff et al., 2016; Marchetti et al., 2016; DuPre and Spreng, 2018). Thus, tracking spontaneous thoughts, especially their sticky form, could potentially contribute to the understanding of the mechanisms underlying depression or the development of a biomarker for rumination useful in depressive relapse detection.

It is worth noting that stickiness and task-relatedness are two orthogonal dimensions of spontaneous thoughts, a sticky thought can be either task-related or task-unrelated. While mind-wandering is concerned with the direction of spontaneous thought, the stickiness of thought is concerned with the mode of spontaneous thought and its dynamics. Despite substantial debates about the appropriate definitions of spontaneous thought (Andrews-Hanna et al., 2014; Smallwood and Schooler, 2015), the measurement of spontaneous thought is based on quite some consensus. Specifically, spontaneous thought is measured by inserting so-called “thought probes” into psychological tasks—questions that ask the participant to report on the nature and contents of their thoughts at the moment the thought probe appears. The stickiness of thoughts can be derived from participants’ responses on a Likert scale to the question of how difficult it was to disengage from the thought. In the task-focused situation, high stickiness reflects a participant being completely engrossed in the task (van Vugt and Broers, 2016), whereas in the mind-wandering situation, high stickiness reflects a participant being completely absorbed in task-unrelated thought, which are likely to be concerns or worries (Marchetti et al., 2016). In contrast, mind-wandering is always defined in terms of whether one is paying attention to the task or to something else (McVay and Kane, 2012b; Smallwood and Schooler, 2015).

A task that has been used frequently to assess off-task thinking is the Sustained Attention to Response Task (SART). The SART consists of thought probes embedded in a go/no-go task, in which participants are asked to press the button as quickly as possible for lower-case words (non-target “Go” trials) while withholding the response for upper-case words (target “Nogo” trials). Studies have found that task performance tends to be worse prior to off-task reports compared to on-task reports (Kam et al., 2011). Similarly, lower accuracy on the sustained attention to response task (SART) has been associated with off-task thinking (McVay and Kane, 2009, 2013). Performance has also been shown to be worse prior to reports of more sticky thought compared to reports of less sticky thought (van Vugt and Broers, 2016).

Of relevance for tracking spontaneous thoughts in real time is that this process tends to be accompanied by decoupling from perceptual input: when attention is directed to internal experiences, the processing of external input is reduced (Smallwood et al., 2008; Schooler et al., 2011). Evidence for such perceptual decoupling has been found in various neural and physiological measures. In an fMRI study, mind-wandering was associated with reduced activation of the occipital cortex, which is likely to reflect a reduction in perceptual processing as well (Gorgolewski et al., 2014). An fNIRS study found that mind-wandering was characterized by significant activations over the medial prefrontal cortex (Durantin et al., 2015), a brain region within the Default Mode Network (DMN, which further includes the posterior cingulate cortex and the medial temporal lobe). The Default Mode Network is a set of brain areas that commonly activates during periods of rest, but also during mind-wandering. This DMN activation during mind-wandering is thought to reflect the active generation of trains of thought (Mantini et al., 2007; Hlinka et al., 2010). Mind-wandering state has also been linked with a smaller pupil response relative to when the person was on-task (Mittner et al., 2014; Unsworth and Robison, 2016; Konishi et al., 2017). Moreover, we have previously shown that when thoughts are considered to be difficult to disengage from by the participant, they are accompanied by greater reductions in pupil size compared to when they are easier to disengage from (Huijser et al., 2020).

Evidence for perceptual decoupling during spontaneous thought is also visible in electrophysiological data. Studies using EEG showed attenuated P1 and N1 event-related-potentials (ERPs) during self-reported mind-wandering (Kam and Handy, 2013; Baird et al., 2014; Broadway et al., 2015; Denkova et al., 2018). As both P1 and N1 components are usually considered to be indicators of early sensory processing, the reduction in P1 and N1 may reflect an attentional attenuation of sensory input (Baird et al., 2014). The effect of mind-wandering was observed not only in neural correlates of perceptual processing but also in event-related potentials associated with a later stage of processing, as demonstrated by studies that demonstrated an attenuated P3 amplitude during mind-wandering (Kam and Handy, 2013; Kam et al., 2015).

Apart from event-related potentials, EEG studies also have reported changes in brain oscillations during mind-wandering. During mind-wandering, there have been reports of higher alpha oscillations (8.5–12 Hz) at posterior occipital sites (Jann et al., 2010; Mo et al., 2013), lower theta oscillations (4–8 Hz) at parietal sites (Jann et al., 2010) and decreased beta oscillations (14–28 Hz) over parietal electrodes (Hlinka et al., 2010). These brain oscillations have been found to some what correlated with DMN activation observed in fMRI studies (Mantini et al., 2007; Hlinka et al., 2010; Jann et al., 2010). It is worth noting that there is to our knowledge no data yet on the electrophysiological changes (including the ERPs and brain oscillations) caused by the sticky form of spontaneous thoughts, despite the behavioral and ocular evidence of increased sensory decoupling when the thoughts are more sticky compared to those which are less sticky (Huijser et al., 2020).

While several studies have demonstrated average differences in the brain or physiological activity between mind-wandering and on-task states, an important question is whether such a signal can be used to predict mind-wandering, and more generally the nature of spontaneous thought, on a single-trial level. Bixler and D’Mello (2016) used a set of eye-gaze features recorded by an eye tracker to predict mind-wandering episodes within 4–10 s before the thought probes during a reading task, and achieved an accuracy of 72% with a Bayes net classifier in a *post hoc* analysis. This number reached 76% in another study, which used oculometric measures including pupil size for the classification of mind-wandering epochs defined as 10 s before the thought probeduring a breath-focusing task (Grandchamp et al., 2014). Combining off-line pupillary and brain activity measures (including the activation of DMN derived from fMRI) to predict single trials of mind-wandering (Mittner et al., 2014) achieved an accuracy of 79.7%. Nevertheless, fMRI is very expensive and not very usable in most real-world contexts. A method that is lower-cost and less invasive is EEG. Jin et al. (2019) used off-line EEG in combination with an SVM classifier to predict mind-wandering trials which were six trials before the thought probes (where for each trial a window of 400 ms before stimulus onset until 1,200 ms after was used), and achieved an accuracy of 60% (Jin et al., 2019). While this accuracy was substantially lower than that of Mittner et al. (2014), it should be mentioned that this classification was task-independent: it could generalize from a SART to a visual search task. A follow-up study showed similar accuracies, but for a classifier that not only generalized across tasks but also across individuals (Jin et al., 2020).

Although the results discussed above show it is possible to predict mind-wandering at an above-chance level using EEG, the prediction accuracy is relatively low. One possible reason for such low accuracy could be that previous studies have not identified the optimal EEG biomarker of spontaneous thought. A potential candidate for such an EEG measure that to date has not been used in EEG studies of spontaneous thought is the steady-state visual evoked potential (Joon Kim et al., 2007; Andersen et al., 2008; Toffanin et al., 2009). This measure is interesting because it is commonly used in brain computer interfaces (BCIs) as a real-time marker of where a participant’s attention is directed (Putze et al., 2019), and therefore is likely to be reliable enough for single-trial prediction of whether a participant pays attention to the task or not.

The SSVEP is the EEG response evoked by visual stimuli that are presented at a specific frequency, which results in an increase in the EEG at that same frequency. When participants direct their attention to these stimuli, this increases the EEG response at the stimulus frequency (Joon Kim et al., 2007). This has been used in many studies of attention (Müller et al., 1998a, b; Toffanin et al., 2009), and also in other studies that sought to differentiate tasks and conditions (Işcan et al., 2011; Wang et al., 2015; Evain et al., 2017; Işcan and Nikulin, 2018). For instance, a study used the SSVEP to find where on the screen a participant was looking by presenting 12 visual target stimuli flickering at different frequencies; classifiers using spatial filtering which was based on canonical correlation analysis (CCA) achieved an average accuracy of 92.78% (Nakanishi et al., 2015). In a visual divided-attention task with targets and distractors presented simultaneously in the left and right visual field, Toffanin et al. (2009) found that the SSVEP had the largest amplitude in a focused-attention condition, intermediate in a divided-attention condition and lowest in an ignored-attention condition (Toffanin et al., 2009). Moreover, the SSVEP technique has also been widely used to investigate high-level processes such as face perception and working memory (Norcia et al., 2015). For example, Ales et al. (2012) flickered their face stimuli that were embedded in scrambled background at a specific temporal frequency while their visibility varied progressively. Compared to previous ERP studies which detected ERP components such as the N170 on the basis of different subjective criteria for the polarity, peak latency, and amplitude, and topography, their SSVEP-based method provided a more objective and quantitative way to measure face detection threshold in terms of the SSVEP response at the flickering frequency (Ales et al., 2012). In another study, stimuli with distinct presentation frequencies were encoded in a working memory task and the SSVEP amplitude was found to be larger for remembered than for forgotten items (Peterson et al., 2014).

Further demonstrating its potential as a neural marker in individual trials, the SSVEP is commonly used in BCI studies as it tends to be robust to perturbations where the subjects were speaking, thinking, or listening in different tasks (Işcan and Nikulin, 2018) and is sufficiently specific in space and time to control an avatar in a virtual environment (Faller et al., 2010). Chen et al. (2015) designed an SSVEP-based speller, which obtained reasonable spelling accuracy (91.04%) and speed (Chen et al., 2015). The SSVEP has even been used in an online home appliance control system, where the four SSVEP stimuli each had their own frequency. This system reached an average accuracy of 92.8% in this online four-way classification (Park et al., 2020).

Taken together, these studies have demonstrated that SSVEP can be used to classify the locus of attention, which by extension suggests that they may be used to indicate the absence of attention towards the ongoing task during mind-wandering, as well as more generally spontaneous thoughts, occur. There are indeed a limited number of studies applying the SSVEP to distinguish between internal and externally directed attention (Vortmann et al., 2021). Even more, virtually no previous studies of the SSVEP have used self-report measures of mind-wandering. The closest we could find was a single study that assessed a concept somewhat related to mind-wandering, which employed a rapid serial visual presentation detection task in which participants were asked to monitor a stream of images presented at 10 Hz for the occurrence of a target image (Macdonald et al., 2011). After every trial, the experimenters asked participants to rate their attentional state on a continuous scale ranging from “fully absent” to “sure present”. The idea was that attentional disengagement should be accompanied by an increase in 10 Hz activity. Results suggested that the subjective attentional state was related to the amplitude of pre-stimulus alpha power (8–12 Hz) instead of the 10 Hz SSVEP. Specifically, during attentional disengagement (presumably similar to mind-wandering), alpha power was significantly higher (Macdonald et al., 2011).

This single study by Macdonald and colleagues does not allow us to draw strong conclusions about the relation between SSVEP amplitude and mind-wandering for several reasons. First, the subjective reports of attentional state in Macdonald may not have reflected mind-wandering because the content of thoughts was not explicitly reported. Hence, it is possible that the states labeled as “low attention” reflected distraction by external stimuli rather than internally-directed mind-wandering. Secondly, in contrast to other studies measuring mind-wandering, including measurements in daily life (34%–47%), educational environment (33%–41%), and lab environment (over 40%; Killingsworth and Gilbert, 2010; McVay and Kane, 2013; Seli, 2016; Wammes et al., 2016; Groot et al., 2021), the occurrence of low attentional state was relatively low (25% of the trials) in the study by Macdonald. We speculate this could be due to the RSVP task they used is a highly demanding task with limited periods to wait for a target, and consequently, there is little time for the mind to wander compared to the SART which has been typically used in previous studies. This might be further compounded by the fact that ratings were requested every trial, which could have brought the participant back to focus.

In summary, the electrophysiological difference between more sticky and less sticky moments of thinking has so far remained largely unexplored. In addition, while work with an RSVP task suggests the SSVEP can contribute to the tracking of mind-wandering, it is not clear whether that generalizes to a more commonly-used SART task, and whether it can help to track stickiness of thought. To fill these gaps, we will employ a SART task—a low-cognitive control task that is commonly used in mind-wandering research—in which we induce an SSVEP by flickering the stimuli to explore whether the SSVEP can contribute to detecting a sticky form of spontaneous thinking as well as mind-wandering.

## Method

### Design

We used a within-subject design to examine whether the SSVEP can be used to track spontaneous thoughts. This was done by asking participants to perform a sustained attention to response task, and comparing the amplitude of the SSVEP between subjectively less sticky trials vs. more sticky trials, and self-reported “on-task” trials vs. “mind-wandering” trials. These self-reports were derived from thought probes that were inserted into the task with intervals varying randomly between 30 and 90 s.

### Participants

Forty participants (25 female and 15 male) with normal or corrected-to-normal vision who were proficient in English were recruited for the experiment through social media. We recruited participants without a history of epilepsy to reduce the possibility that seizures could be triggered by the flickering stimuli embedded in our task. The research proposal was approved by the Research Ethics Committee (CETO) of the Faculty of Arts, University of Groningen. Participants gave informed consent, and they were paid 24 euros for their participation in the whole experiment which lasted approximately 2.5 h.

### Questionnaires

In an effort to investigate how the occurrence of mind-wandering varies for people who show different vulnerabilities to ruminative thought and depression, all prospective participants were asked to fill out a number of questionnaires. These questionnaires are irrelevant to the current research question and will be elaborated on in a separate report. The questionnaires we used to determine vulnerability to ruminative thought are the Perseverative Thinking Questionnaire (PTQ) measuring repetitive negative thinking (Ehring et al., 2011), Rumination Response Scale (RRS) for accessing depressive rumination (Nolen-Hoeksema and Morrow, 1991), and the CES-D indicating the severity of depression (Radloff, 1977). To be able to select participants based on a single score, the total score (X) of the three questionnaires was calculated as the sum of the separately standardized score of each form: $X=\sum \frac{\left(x_{i}-{\bar{x}}_{i}\right)}{\sigma_{i}}$, where *x* was the raw score, σ was the standard deviation and *i* ranged from 1 to 3. Participants within the top and bottom 25% of the distribution of the total score were selected for the laboratory experiment. The average and standard deviation of the PTQ, RRS, and CES-D scores for the high and low depression groups are shown in Table 1.

**Table 1:** The average and standard deviation (in brackets) of the scores across the high and low depression groups.

**Group**	**Questionnaires**			**Writing manipulation^1^**	
	**PTQ score**	**RRS score**	**CES-D score**	**Intensity**	**Frequency**
High depression	45.85 (4.70)	67.05 (7.34)	51.65 (6.47)	4.76 (0.97)	4.35 (0.86)
Low depression	25.10 (8.33)	41.75 (8.34)	35.45 (3.71)	4.11 (0.74)	3.84 (0.83)
Difference between groups	*t*_(38)_ = 9.70, *p* < 0.001	*t*_(38)_ = 10.19, *p* < 0.001	*t*_(38)_ = 9.71, *p* < 0.001	*t*_(34)_ = 2.31, *p* = 0.027	*t*_(34)_ = 1.81, *p* = 0.08

^1^Due to incomplete filling in of the writing manipulation, we are missing three data points in the high depression group and one data point in the low depression group.

When they passed the screening, participants were required to complete an additional set of questionnaires online, approximately two days before the experiment: (1) Cognitive Failures Questionnaire—Memory and Attention Lapses (CFQ-MAL; McVay et al., 2009; McVay and Kane, 2013); (2) Personal Concerns Inventory (PCI; Miles Cox and Klinger, 2008; McVay and Kane, 2013); (3) Adult ADHD Self-Report Scale (McVay and Kane, 2013); and (4) Action Orientation Scale (AOS; Kuhl, 1985). We used the PCI to extract participants’ worries and concerns (McVay and Kane, 2013). From the PCI, four of the most important worries and concerns (according to the participants’ ratings) were selected and converted into word triplets (e.g., if the participant reported being worried about talking to strangers, we converted this into “easy-communication-stranger”), which were embedded in the SART task unbeknownst to the participant to induce mind-wandering episodes (McVay and Kane, 2013). The other three questionnaires were used as filler questionnaires.

### Procedure

#### Writing manipulation

To further increase the probability of mind-wandering, participants were asked to complete a writing task about a negative event in their life just prior to starting the SART experiment. This task was chosen because it is known that rumination about negative events tends to generate mind-wandering (Miles Cox and Klinger, 2008), especially thoughts of the sticky type. Participants were given 10 min to describe this event (for the text of the instructions, please refer to Appendix, derived from Banks et al. (2019). After the completion of the writing assignment, participants were asked to indicate on a 6-point Likert Scale how strongly this event influenced them and how frequently it bothered them (Banks et al., 2019). The average and standard deviance of the scores (intensity and frequency) are also presented in Table 1.

#### SART

**Figure 2:**
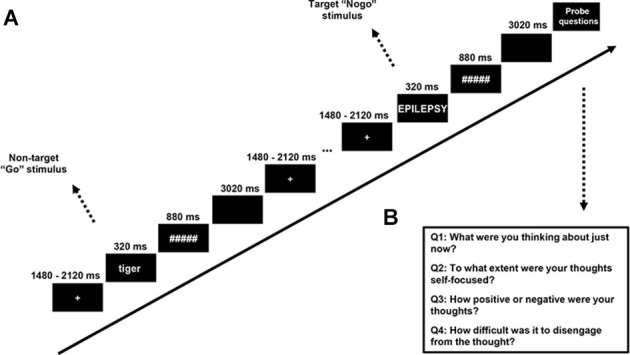
**(A)** Stimuli and procedure of the SART—all stimuli were flickering with a frequency of 12.5 Hz. A fixation cross was present firstly for a variable duration (sampling from 1,480, 1,640, 1,800, 1,960, or 2,120 ms). Word stimuli were presented for 320 ms and a mask presented for 880 ms followed. A trial ended with a 3,020-ms blank screen. **(B)** Thought probe questions.

To measure the SSVEP, we developed a “flicker” version of SART, in which all the stimuli except the thought probes were presented while appearing and disappearing at a rate of 12.5 Hz (i.e., switching from “on” to “off” every 80 ms). This frequency was chosen to evoke a high level of SSVEP response, which is known to be better for lower frequencies (Norcia et al., 2015) while avoiding the contamination of alpha band oscillation (8–12 Hz). A trial in the SART consisted of 320 ms flickering words in the center of the screen following a flickering cross of 200*200 pixels, which was itself presented for an average duration of 1,800 ms (jittered with a period ranging from 1,480 ms to 2,120 ms to reduce expectancy effects in brain and behavior). The words were followed by a flickering mask for 880 ms, then an interstimulus interval (ISI) of 3,020 ms without any flickering^1^, after which the next trial began (see Figure 2 for the procedure of the SART). Word stimuli for each trial were generated in a pre-defined order from a word list derived from McVay and Kane (2013). Following McVay and Kane, designated trials consisted of the words created on the basis of participants’ answers to the PCI. Participants were asked to press the button “N” as quickly as possible for lowercase words (No-target “Go” trials, which made up 88.89% of the trials) and to withhold their response for uppercase words (Target “Nogo” trials, which made up 11.11% of the trials). The word triplets were presented five trials before the target trial, which was immediately followed by a thought probe. The SART contained eight blocks, each block included 90 trials and six thought probes (self-report questions about the mental state of the participant).

The task was presented on a computer, and administered *via* Psychopy software (Peirce et al., 2019), with a viewing distance of 65 cm away from the screen. The refresh rate of the screen was set to 50 Hz which is four times the flickering frequency, allowing us to generate 12.5 Hz flickers perfectly. The visual angle of the word stimuli ranged from 5.72 degrees to 36.39 degrees horizontally (depending on word length), and 5.55 degrees vertically.

The thought probe used to determine the content of participants’ thought followed the format of our previous studies on mind-wandering (Huijser et al., 2018; Jin et al., 2019). Specifically, self-reported thought content was measured with the question “What were you thinking about just now?” Participants had to select among the options: (1) I was completely focused on the task; (2) I was evaluating aspects of the task. (e.g., how I was doing or how long the task was taking); (3) I was thinking about personal things; (4) I was distracted by my environment. (e.g., sound, temperature, my physical state); (5) I was daydreaming or thinking about task-irrelevant things; and (6) I was not paying attention and did not think about anything in particular. To ensure a full understanding of the thought probes, participants were asked to give an example of each option to the experimenter and a practice session was run before the experiment began where participants could have any question answered. Consistent with our previous study, the responses to the thought probes were designed to be categorical instead of binary or a Likert scale ranging from on-task to mind-wandering. This methodology allowed us to obtain an impression of thought content while avoiding the acquiescence bias inherent in the binary response (Weinstein, 2018). Trials with a response of: (1) completely focused on the task and; (2) evaluating the task were classified as “on-task” while trials with a response of; (3) thinking about personal things and; (4) daydreaming or thinking about task-irrelevant things were classified as “mind-wandering”. The answers of (5) distracted by the environment; and (6) not thinking anything, in particular, were not considered for further analysis as they could not be clearly assigned to either mind-wandering or on-task.

To index stickiness, we used an additional question: “How difficult was it to disengage from the thought?”, scored on a scale from 1 (very easy) to 9 (very difficult). Trials with responses ranging from 1 to 4 on the 9-point rating scale were classified as “less sticky” trials while responses ranging from 6 to 9 were classified as “more sticky” trials. Trials with the intermediate response of 5 were excluded from further analysis because they could not be assigned to either category. This concerned 19.38% of the responses. We think it is justified to dichotomize responses in this way because participants were instructed that the response “5” was neither non-sticky nor sticky, and therefore clearly bisects the scale. Dichotomization was necessary since considering each point of response independently would result in too few responses per category. This was compounded by the fact that more often than not, the participants did not use every single ordinal response which made the conclusion limited when comparing the SSVEP for each point of response.

Apart from this, we followed previous mind-wandering studies by asking participants to report on the degree of self-focus, which was measured with the question “To what extent were your thoughts self-focused?”, scored on a scale from 1 (completely not self-focused/about others) to 9 (completely self-focused). The valence of the thoughts was measured with the question “How positive or negative were your thoughts?”, scored on a scale from 1 (very negative) to 9 (very positive). All the self-reported labels derived from the thought probes (task-unrelated thoughts, self-focus, positiveness, and stickiness) were assigned to the five trials preceding the thought probe for the subsequent statistical comparison and classification. In this study, only the task-relatedness of self-reported thought and the stickiness of the thoughts will be reported.

It is worth noting that the task-relatedness and the stickiness of the thoughts are separate dimensions of self-reported thoughts, each trial is marked with both labels separately in this study. In case the participants were on-task and unsure about how to rate their current level of stickiness, they were instructed to rate their thoughts as neither “very difficult” nor “very easy” to disengage from (corresponding to a medium level of stickiness which was around 5 in a 9-point rating scale, and these labels were excluded from further analysis).

### EEG Recording

EEG was recorded with a sampling rate of 512 Hz by a Biosemi 32-channel system (BioSemi, Amsterdam, Netherlands) with six individual electrodes to measure eye movements and mastoid signals. An electrode near the vertex was used as the on-line reference. We ensured that for all participants, all impedances were kept below 20 kΩ.

Before entering the session of SART, a 5-min resting state EEG was recorded (not reported in this manuscript). The instructions for the resting state were the same as in a previous study on mind-wandering (Diaz et al., 2014). During these 5 min, participants were asked to stay relaxed and quiet, to keep their eyes closed, and not fall asleep. The resting period started as they pressed the space button and immediately closed their eyes. A short sound was played by a speaker indicating the end of the 5-min resting phase. Participants were told that if they failed to follow the instructions and opened their eyes before the phase ended, they would be asked to restart the resting state recording. No participant failed to follow these instructions.

### EEG Analysis

#### EEG pre-processing

The EEG data were pre-processed with the EEGLAB toolbox (Delorme and Makeig, 2004) and MATLAB (The MathWorks, Inc., Natick, MA). EEG signals were band-pass filtered (0.5–40 Hz), followed by epoching the continuous data into trials from 500 ms before stimulus onset to 4,300 ms after. The EEG data were baseline-corrected to the time period from 500 ms before the onset to the stimulus onset time. Following this, the period from −500 ms before stimulus onset time to 1,300 ms after was used for further analysis. Trials with artifacts due to head and muscle movements as well as those containing slow drifts were removed from further analysis by visually inspecting all the trials. Bad channels were detected for two participants *via* visual inspection and replaced through interpolation of adjacent electrodes, and for each of these participants, only one electrode was replaced. Data were re-referenced to the average signal from all electrodes. An independent component analysis (ICA) was performed to remove ocular artifacts. Due to the interpolation of bad channels and the average referencing, the rank of the data was detected and adjusted automatically by the pop_runica function when running the ICA. The independent components that were considered to be related to eye blinks, muscle activity, and horizontal saccades were removed after visual inspection by an experienced rater (a maximum of five out of 38 components were removed). Time points from 200 ms before the stimulus and 900 ms after the stimulus was further selected for each trial, followed by a baseline correction to ensure that the EEG waveforms were centered on zero again after the data had been segmented from longer epochs. The time window of the segmentation was decided based on the moment when the ERP returned back to baseline, which was at 900 ms (see Figure 4). The pre-processing removed an average of 12.72% (ranging from 2.14% to 43.59%) of the trials that were used in the subsequent analysis because they were part of the five trials before thought probes. The trials were labeled (as mind-wandering/on-task and more sticky/less sticky) after the pre-processing to avoid subjective bias during the manual artifact removal. Among a total of 240 trials per participant, an average of 88.15 trials of on-task and 77.35 trials of mind-wandering remained after the pre-processing, while an average of 87.65 more sticky trials and 76.68 less sticky trials were retained.

**Figure 3:**
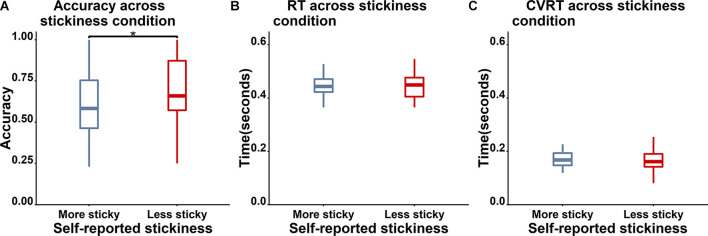
Boxplot of behavioral performance differences between less sticky trials (red) and more sticky trials (blue). **(A)** Accuracy for Nogo trials is significantly higher for less sticky trials (“*” indicates a p value which is lower than 0.05). **(B)** and **(C)** No significant differences between more and less sticky trials were observed in RT and cvRT of Go trials.

**Figure 4:**
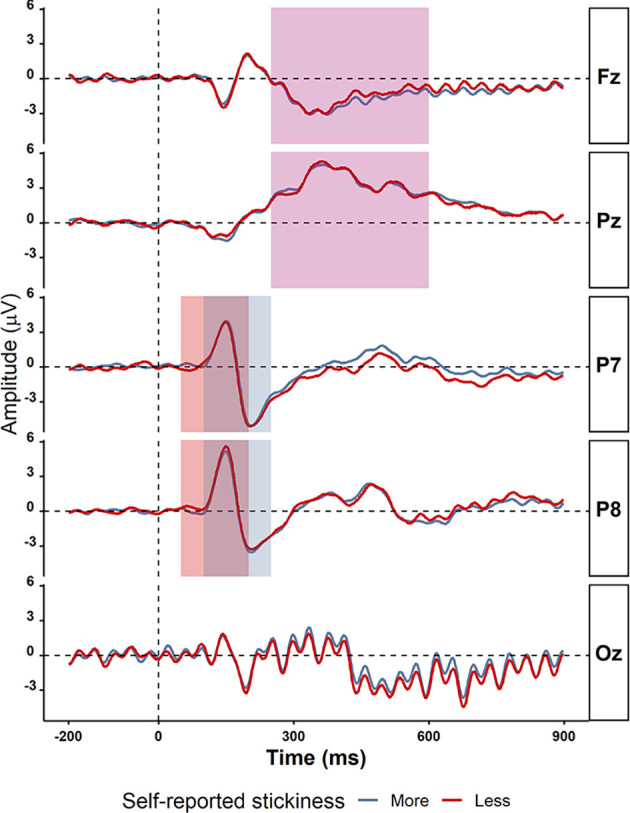
Grand average ERPs in electrodes P7, P8, Fz, Pz, and Oz comparing more sticky trials (red) and less sticky trials (blue). The time windows for detecting peaks of ERPs components are shown in the rectangles: P1 (50–200 ms in light green), N1 (100–250 ms in light purple), and P3 (250−600 ms in light yellow). There were no significant differences between more and less sticky trials.

#### Event-related potential (ERP) analysis

After obtaining clean data, the on-task and off-task trials were averaged separately. The P1 and N1 component was investigated in P7 and P8 electrodes as was done in other ERPs studies (di Russo et al., 2019), while the P3 component verbed in Pz and Fz electrodes; following (Denkova et al., 2018; Gonçalves et al., 2018; Maillet et al., 2020). A time window of 50–200 ms was selected for detecting the peak amplitude of P1, 100–250 ms for N1, and 250–600 ms for P3. The time windows were decided based on prior studies but slightly enlarged after checking the ERP plot for all participants to make sure the peak latencies were within the time window range (Jin et al., 2019). The peak amplitudes were calculated as the highest (for P1 and P3) or lowest (for N1) amplitudes during the corresponding time windows.

#### Frequency analysis

The oscillatory power analysis for single trials used the same methodology as our previous study (Jin et al., 2019). EEG epochs in electrodes P7, P8, Pz, and Fz were filtered using a filter kernel constructed with firls in MATLAB (The MathWorks, Inc.), and decomposed into alpha (8–12 Hz) and theta (4–8 Hz) frequency bands. The four electrodes were selected based on the fact that they showed effects of mind-wandering in alpha and theta bands in previous studies (Jin et al., 2019): the filter order was set as EEG sampling rate divided by the lower bound of the frequency band, i.e., four for theta and eight for alpha. The finite response filter was defined as a plateau-shaped vector [0 0 1 1 0 0] where 1 reflects the frequency range in the band and 0 reflects the surrounding frequencies (Cohen, 2014). A transition width of 0.2 was used for the kernel, which is a somewhat intermediate trade-off between time- and frequency resolution. After applying the filter with the filtfilt function, the EEG signal was Hilbert-transformed to obtain the phase angles in the complex plane. These complex numbers were transformed into estimates of the power spectral density (PSD) using pwelch function. Power estimates were computed for the pre-stimulus and the post-stimulus intervals separately. The power value was calculated as the square of the absolute value of PSD.

#### Inter-trial coherence analysis

To measure the EEG response to the flickering stimuli across frequencies, inter-trial coherence (ITC) was computed. The ITC is also known as “phase locking value”, ranging from 0 which represents completely random activity across trials, to 1 which represents completely phase-locked activity across trials. The calculation of ITC and event-related spectral perturbation (ERSP) was implemented in EEGLAB and MATLAB (The MathWorks, Inc.) with the newtimef function which used a frequency spacing of 0.5 Hz in the frequency range from 8 to 30 Hz for time-frequency decomposition. The number of cycles used by the wavelet decomposition varied from 5 cycles at the frequency of 8 Hz to 9.375 cycles at the frequency of 30 Hz. As the SSVEP has commonly been observed in the occipital region of the brain (Kastner-Dorn et al., 2018), the Oz electrode was used to track the ITC value in this study. A time window from 500 ms to 900 ms was selected since the SSVEP requires some time (around 500 ms) to reach its maximum after the onset of the flicker, and the SSVEP waveform at Oz takes around 900 ms to return to baseline level (Müller et al., 1998b). To evaluate the ITC at the flickering frequency (12.5 Hz) and its harmonics at 25 Hz, we computed the mean ITC of 12–13 Hz and 24.5–25.5 Hz separately.

#### Event-related spectral perturbation (ERSP) analysis

The ERSP of the single trials was computed on the same frequency ranges as the ITC: the flickering frequency (12–13 Hz), the harmonic frequency (24.5–25.5 Hz), and the alpha band frequency (8–12 Hz) within the time window ranging from 500 ms to 900 ms after the stimulus onset.

#### Single-trial ERP analysis

In previous studies, ERPs components including N1, P1, and P3 were associated with mind-wandering and they were the potential predictors of mental states (Braboszcz and Delorme, 2011; Broadway et al., 2015; Baldwin et al., 2017). To allow for reliable estimates of these ERPs on single trials, which is particularly important for our classifier, we computed single-trial ERPs using methods used in our prior studies (Jin et al., 2019, 2020). The essence of this method is template matching. First, it builds an ideal ERP template with a pre-defined Mexican hat function (Bostanov and Kotchoubey, 2006) and then computes its cross-covariance with the single trial EEG. After detecting the local extrema of the points that best match between the signal and the template in the time domain, the resulting features are the amplitude W, the time lag t, and the scale s for each component in each trial. A more detailed description of the single-trial ERP methodology can be found in Bostanov (2015) and Jin et al. (2019).

Apart from the single-trial ERP components, the alpha and theta oscillations, as well as the ERSP, were also obtained for every single trial using the methods described above in section “EEG Analysis”. These features were fed to the classifier for prediction.

### Classifier of spontaneous thought with neural data

After obtaining all the features, we were interested in whether the SSVEP would contribute to the accuracy of classification. Two classifiers built on the basis of all the above-mentioned features with and without the SSVEP were designed, using the same methods as Jin et al. (2019). We deliberately did not search for additional features but instead restricted ourselves to EEG features that had previously been related to mind-wandering, and that had been reported in our prior studies in which we classified mind-wandering. The latter was important so we could compare our results of the experiment that included the SSVEP to these previous studies that did not include the SSVEP.

To classify whether the participant was mind-wandering or on-task for each trial, an SVM was implemented with caret package (Kuhn, 2008) in R. Before starting classification, features were normalized by calculating *Z*-scores across trials for training and testing datasets separately within each participant. The whole dataset was randomly divided into train and test datasets, for which the proportion of mind-wandering and on-task trials remained the same as in the original dataset (70% of the data was allocated to training and 30% was allocated to testing). We used 10-fold cross-validation to train the classifier (Fushiki, 2011). Since there were unequal numbers of trials in the on-task and mind-wandering states, an oversampling method was implemented for each fold in an effort to make sure the number of mind-wandering and on-task trials was equal in the training dataset (Gosain and Sardana, 2017). A radial basis function (RBF) was used as a kernel on the SVM classifier, as it has been shown to be suitable for classifying high-dimensional data including EEG (Subasi and Ismail Gursoy, 2010; Li et al., 2018) and was effective in our previous mind-wandering study (Jin et al., 2019). Kernel parameters that could be adjusted were the cost C of the radial kernel which controls the complexity of the boundary between support vectors, and the smoothing parameter sigma (also known as gamma). Both parameters were tuned for each participant using grid search optimizing accuracy with sigma ranging from 0.001 to 10 and C ranging from 0.00001 to 0.1 (Liu et al., 2012; Ravi Kumar et al., 2020). Tune length, i.e., the amount of granularity in the tuning parameter grid, was set to 9 for all participants. To evaluate whether the SSVEP improved the classifier, we compared the accuracies of the classifiers including and not including the SSVEP. In addition to reporting accuracy, we report sensitivity and specificity to be able to assess bias. Sensitivity and specificity were calculated as follows: sensitivity = TP / (TP + FN); specificity = TN / (TN + FP) while TP represents the true positives, TN represents the true negatives, FP represents the false positives and FN represents the false negatives (van Stralen et al., 2009).

### Statistics

The comparisons across conditions (mind-wandering vs. on-task, more sticky vs. less sticky) over the behavioral results, ERP components, alpha and theta oscillations, ITC and ERSP of 12.5 Hz and 25 Hz were done by means of a paired t-test (using the t.test function in R; R Core Team, 2020) as well as by means of the calculation of pairwise Bayes factors (using the ttest BF function in the BayesFactor package; Morey et al., 2011). Our motivation for adding Bayes Factors (*BF*_10_) is that in the case of null results, those allow us to distinguish between general uncertainty in our data and evidence for the null hypothesis (0) over the alternative hypothesis (1). In general, a BayesFactor *BF*_10_ between 0.3 and 3 indicates that the data do not allow us to draw any definitive conclusion (i.e., uncertainty), while a BayesFactor *BF*_10_ larger than 3 indicates evidence in favor of the alternative hypothesis, and a BayesFactor *BF*_10_ smaller than 0.3 indicates evidence in favor of the null hypothesis (Morey et al., 2011). To correct for chance level in the classification with small a sample size, the binoinvfunction in MATLAB was used to compute the statistically significant threshold: $St\left(\alpha \right)=binoinv\left(1-\alpha ,\;n,\;\frac{1}{c}\right)\times \frac{100}{n}$, where α is the significance level, n is the sample size and c is the number of classes for prediction (Combrisson and Jerbi, 2015). The code for our data analysis can be found at https://github.com/hankyoung1324/FlickeringSART.

## Results

### Behavioral results

We first examined behavioral performance during the SART. Go-trial accuracy was 99.11% (with a standard deviation, *SD* of 1.31%), while Nogo accuracy was 67.66% (*SD* 17.87%). Participants performed significantly worse for Nogo trials than for Go trials [*t*_(39)_ = 11.27, *p* < 0.001, BayesFactor (*BF*_10_) = 9.61*10_10_ in a paired *t*-test]. The average response time for Go trials was 471 ms (*SD* 84 ms). Here we firstly report the results of stickiness in sections “Behavioral results”–“Tracking Stickiness on Single Trials”, followed by the results of the mind-wandering vs. on-task in section “Tracking Mind-Wandering”.

Among all the 720 trials of the experiment, a total of 236 trials that comprised the five trials before each of the thought probes were selected for further analysis. Of these, an average of 36.58% labeled trials were marked as less sticky while 43.80% trials were marked as more sticky. There were also 19.38% of the trials reported as neutral stickiness and 0.20% with no responses and were not included in the analysis. Nine participants did not use the full range of stickiness ratings, which had the consequence that there were no trials in one of the two categories (more sticky or less sticky). For this reason, these participants were not included for further analysis. The proportions of more sticky/less sticky trials as well as mind-wandering/on-task for each participant are reported in the supplementary Supplementary Table A1.

To examine the validity of the thought probe responses, we firstly asked whether there was a difference in task performance between different responses on the thought probes (see Figure 3 for the boxplot and Table 3 for the descriptive results). We found that the level of stickiness of thoughts was indeed associated with changes in behavior: accuracy for Nogo trials was lower when thoughts were judged to be more sticky (*M* = 0.58, *SD* = 0.2) compared to less sticky (*M* = 0.68, *SD* = 0.22) moments (*t*_(30)_ = 3.06, *p* = 0.005, *BF*_10_ = 8.54), yet the stickiness did not affect RT (More sticky: *M* = 0.47, *SD* = 0.10 and less sticky: *M* = 0.48, *SD* = 0.11, *t*_(30)_ = 0.18, *p* = 0.86, *BF*_10_ = 0.19), nor did it affect the coefficient of variation of RT (cvRT, i.e., standard deviation of RT/ mean RT):More sticky: *M* = 0.26, *SD* = 0.25 and less sticky: *M* = 0.27, *SD* = 0.27, *t*_(30)_ = 0.23, *p* = 0.86, *BF*_10_ = 0.20; see Figure 3 for the boxplot and Table 3 for descriptive results).

**Table 2:** The accuracy, sensitivity, and specificity of the support vector machine model with and without the ERSP value included.

**Comparison**	**With or without ERSP**	**Accuracy**	**Sensitivity**	**Specificity**
More sticky vs. less sticky trials	Without ERSP	0.73	0.32	0.68
	With ERSP	0.73	0.35	0.66
Mind-wandering vs. on-task trials	Without ERSP	0.64	0.42	0.57
	With ERSP	0.64	0.45	0.54

**Table 3:** Task performance of the “Nogo” trials as a function of responses on the different thought probes.

**Thought probe**	**Types**	**Accuracy**	**RT (seconds)**	**cvRT (seconds)**
Task-relatedness	Mind-wandering	0.62 (0.21)	0.41 (0.04)	0.17 (0.09)
	On-task	0.69 (0.23)	0.51 (0.59)	0.14 (0.10)
Self-focus	More self-focused	0.61 (0.21)	0.43 (0.12)	0.20 (0.24)
	Less self-focused	0.62 (0.26)	0.42 (0.08)	0.13 (0.09)
Positive or negative	Positive	0.69 (0.27)	0.45 (0.14)	0.21 (0.29)
	Negative	0.52 (0.21)	0.41 (0.07)	0.20 (0.24)
Stickiness	More sticky	0.59 (0.20)	0.43 (0.07)	0.16 (0.08)
	Less sticky	0.69 (0.22)	0.53 (0.67)	0.16 (0.25)

The bracket includes the standard deviation (*SD*).

### EEG

#### ERPs

We then asked whether—even though there were no behavioral differences between more sticky and less sticky trials—there were significant differences between more sticky and less sticky trials in the amplitude of the P1, N1, and P3 event-related potentials (ERPs) as prior studies have shown those to be sensitive to mind-wandering. The average ERPs waveforms from −200 ms to 900 ms were plotted for more sticky and less sticky trials separately as shown in Figure 4. Note that these ERPs exhibit strong periodicity occipitally due to the flickering of the stimuli. To compare more sticky and less sticky trials, we focused on the peak amplitude of the ERP components P1, N1 and P3. For many of these ERPs, their amplitude was the same in trials in which the spontaneous thoughts were more sticky compared to trials that were less sticky (P1 component measured at the P7 electrode: more sticky (*M* = 4.59, *SD* = 2.59) and less sticky (*M* = 5.03, *SD* = 2.26), *t*_(30)_ = 1.01, *p* = 0.32, *BF*_10_ = 0.30; N1 components at the P8 electrode [more sticky (*M* = −4.97, *SD* = 2.86) and less sticky (*M* = −4.98, *SD* = 3.00), *t*_(30)_ = 0.16, *p* = 0.87, *BF*_10_ = 0.19]; P3 component measured at the Pz electrode [more sticky (*M* = 6.34, *SD* = 2.77) and less sticky (*M* = 6.36, *SD* = 2.48), *t*_(30)_ = 1.15, *p* = 0.26, *BF*_10_ = 0.35]. At some electrodes, the difference was unclear [N1 component measured at the P7 electrode (more sticky (*M* = −5.92, *SD* = 2.63) and less sticky (*M* = −6.52, *SD* = 2.62), *t*_(30)_ = 1.67, *p* = 0.11, *BF*_10_ = 0.66)] and P3 components measured at the Fz electrode [more sticky (*M* = 1.34, *SD* = 1.43) and less sticky (*M* = 1.86, *SD* = 1.57), *t*_(30)_ = 2.02, *p* = 0.05, *BF*_10_ = 1.13].

#### Oscillatory power

We then asked whether alpha and theta power differed between more sticky and less sticky trials, since previous studies associated those with the occurrence of spontaneous thought especially mind-wandering. Data were too uncertain to determine whether there was an effect of stickiness on the alpha band power spectrum on posterior electrode Pz [more sticky (*M* = 0.33, *SD* = 0.70) and less sticky (*M* = 0.24, *SD* = 0.44), *t*_(30)_ = 1.36, *p* = 0.19, *BF*_10_ = 0.44, Table 4 gives a complete overview of statistical results for all electrodes]. In contrast, frontal electrode Fz showed no difference in theta power between more and less sticky trials [more sticky (*M* = 0.13, *SD* = 0.25) and less sticky (*M* = 0.10, *SD* = 0.28), *t*_(30)_ = 0.23, *p* = 0.82, *BF*_10_ = 0.20].

**Table 4:** Pairwise t-tests comparing oscillatory power in alpha and theta band at the P7, P8, Fz, and Pz electrodes between different groups of trials.

**Comparison**	**Frequency band**	**Electrod**v	***t* value (t)**	**Degree of freedom (df)**	***p* value (p)**	**Bayes Factor (*BF*_10_)**
More sticky vs. less sticky	Alpha	P7	1.26	30	0.22	0.39
trials		P8	1.14	30	0.26	0.35
		Fz	0.81	30	0.43	0.26
		Pz	1.36	30	0.19	0.44
	Theta	P7	1.51	30	0.14	0.53
		P8	1.18	30	0.25	0.36
		Fz	0.23	30	0.82	0.20
		Pz	1.06	30	0.30	0.32
Mind-wandering vs.	Alpha	P7	1.68	38	0.10	0.62
on-task trials		**P8**	**2.48**	**38**	**0.02**	**2.53**
		**Fz**	**2.67**	**38**	**0.01**	**3.74**
		**Pz**	**2.16**	**38**	**0.04**	**1.37**
	Theta	P7	0.03	38	0.97	0.17
		P8	0.13	38	0.89	0.17
		Fz	0.37	38	0.71	0.18
		Pz	1.40	38	0.17	0.42

Bold font indicates a significant contrast.

#### SSVEP

**Figure 5:**
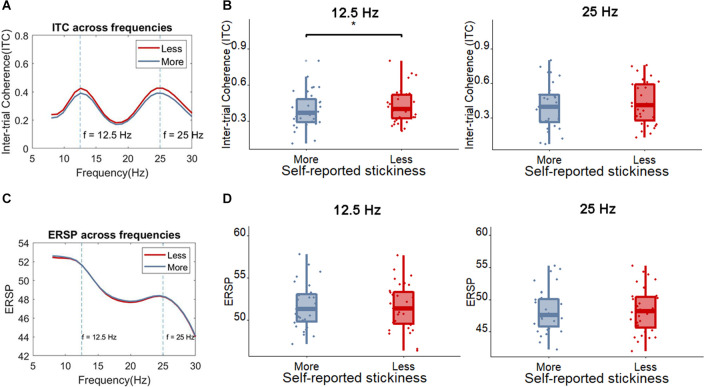
**(A)** The average ITC across frequencies for less sticky and more sticky trials. **(B)** Boxplot of the ITC at the frequency of 12.5 Hz and 25 Hz for less sticky and more sticky trials, each dot represents one participant. **(C)** The average ERSP ((log)dB) across frequencies comparing less sticky and more sticky trials. **(D)** Boxplot of the ERSP at the frequency of 12.5 Hz and 25 Hz for less sticky and more sticky trials, each dot represents one participant. Significant differences are indicated with an *.

Next, we examined whether the SSVEP could distinguish between more sticky spontaneous thoughts and those which were less sticky. The peak SSVEP amplitude was firstly assessed in occipital sites (Oz), the usual locus of the SSVEP. One measure that has been used to quantify the magnitude of the SSVEP is the inter-trial coherence (ITC) at the SSVEP frequency. Figure 5 shows that, as would be expected based on the fact that our stimulus flickers at 12.5 Hz, the peak ITC values occur around 12.5 Hz and 25 Hz in the Oz electrode (where 25 Hz is a harmonic of the flickering frequency). Indeed, the SSVEP measured by means of the ITC showed that less sticky trials showed a slightly higher peak ITC value compared to more sticky trials around 12.5 Hz [more sticky (*M* = 0.39, *SD* = 0.16), less sticky (*M* = 0.42, *SD* = 0.15), *t*_(30)_ = 2.34, *p* = 0.026, *BF*_10_ = 1.99] while around 25 Hz no clear pattern emerged [more sticky (*M* = 0.40, *SD* = 0.20), less sticky (*M* = 0.43, *SD* = 0.19), *t*_(30)_ = 1.41, *p* = 0.17, *BF*_10_ = 0.47]. Meanwhile, no difference was found between more and less sticky trials in the ERSP around the flickering frequency and harmonic frequency of the SSVEP [12.5 Hz: more sticky (*M* = 51.58, *SD* = 2.60), less sticky (*M* = 51.58, *SD* = 2.71), *t*_(30)_ = 0.04, *p* = 0.97, *BF*_10_ = 0.19; 25 Hz: more sticky (*M* = 48.30, *SD* = 3.39), less sticky (*M* = 48.26, *SD* = 3.46), *t*_(30)_ = 0.27, *p* = 0.79, *BF*_10_ = 0.20].

### Tracking stickiness on single trials

**Figure 6:**
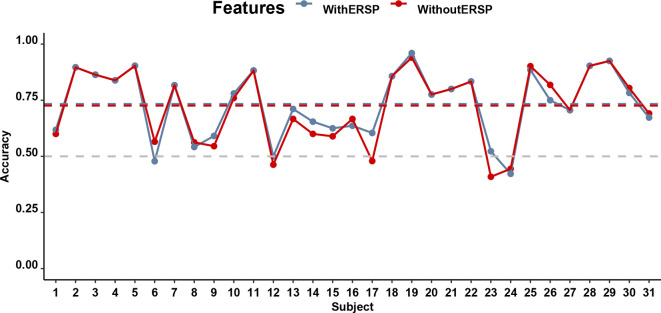
Performance of the classifiers predicting stickiness on the basis of EEG for all participants. The gray horizontal dashed line indicates the chance level of 50% accuracy while the blue and red dashed lines indicate the accuracy of classifiers with and without the ITC, respectively.

Despite the ambiguous statistical differences between low- and high-stickiness trials, it was on a single-trial level possible to predict with an individual-participant classifier based on a multivariate EEG signal whether the participant was engaged in a thought that was sticky or not with accuracies that ranged from 40.90% to 94.00% and an average accuracy of 72.60%. This accuracy did not differ much when ERSP was included, in which case accuracies ranged from 42.22% to 96.00% with an average accuracy of 73.35%. Figure 6 shows the accuracy of the classifiers of the stickiness of thoughts with and without the SSVEP signal, separately for every participant. Both of these accuracies were significantly higher (*p* < 0.01) than the corrected chance level (59.12%) for our sample size (*n* = 159 on average, ranging from 68 to 218 across participants) and the number of classes (2; Combrisson and Jerbi, 2015). The accuracies of the model with and without the ERSP included did not differ from each other (*t*_(30)_ = 0.98, *p* = 0.33, *BF*_10_ = 0.30).

### Tracking mind-wandering

We then examined whether we could predict mind-wandering on a single-trial level. A proportion of 37.92% of the trials was marked as mind-wandering (thinking about personal matters or daydreaming) and 41.80% were focused on the task (completely focused or evaluating aspects of the task), while the remaining 19.73% of the trials were reported uncertain, distracted by the surroundings or the thought was not anywhere specifically. There were also 0.55% of the trials excluded for further analysis as no responses were indicated. One participant was excluded from further analysis as no mind-wandering episodes were reported.

**Figure 7:**
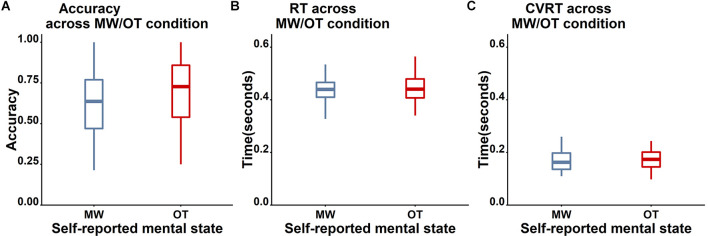
Comparison of the behavioral performance between mind-wandering (MW, blue) and on-task (OT, red) trials. **(A)** Accuracy for Nogo trials. **(B)** RT for Go trials. **(C)** cvRT for Go trials. No clear differences on these three measures were found between self-reported on-task and mind-wandering trials.

Unfortunately, the self-report data were unrelated to task accuracy (see Figure 7 for the boxplot and Table 3 for the descriptive results). Specifically, accuracy on Nogo trials did not differ between mind-wandering (*M* = 0.62, *SD* = 0.21) and on-task (*M* = 0.68, *SD* = 0.23) trials (*t*_(38)_ = 1.71, *p* = 0.096, *BF*_10_ for the paired *t*-test is 0.65 indicating insufficient evidence for any difference). Also RT did not differ between mind-wandering (*M* = 0.47, *SD* = 0.13) and on-task (*M* = 0.46, *SD* = 0.07) trials (*t*_(38)_ = −0.93, *p* = 0.36, *BF*_10_ = 0.26). The same applied to the coefficient of variation of RT (cvRT), which also did not differ between mind-wandering (*M* = 0.27, *SD* = 0.27) and on-task (*M* = 0.25, *SD* = 0.22) trials (cvRT; *t*_(38)_ = 0.39, *p* = 0.70, *BF*_10_ = 0.19).

**Figure 8:**
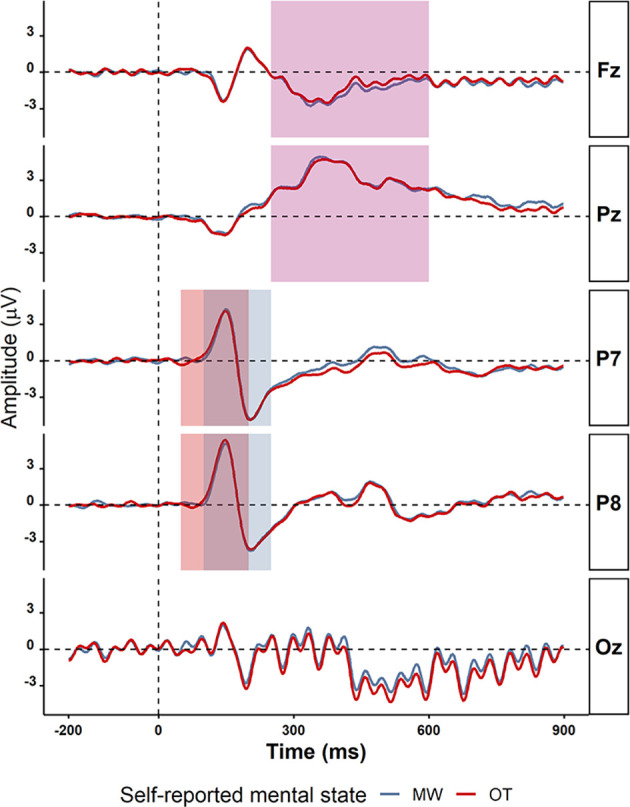
The grand average ERPs on electrodes P7, P8, Fz, Pz, and Oz for mind-wandering trials (MW; blue) and on-task trials (OT; red). The time windows used for detecting the peaks of the various ERP components are shown in different rectangles: P1 (50–200 ms in light green), N1 (100–250 ms in light purple), and P3 (250 −600 ms in light yellow). The electrodes and time windows were selected on the basis of previous studies on mind-wandering.

Alpha power for mind-wandering trials (*M* = 0.41, *SD* = 1.23) was found to be somewhat higher compared to on-task trials (*M* = 0.35, *SD* = 0.97) at posterior sites (alpha in P8: *t*_(38)_ =2.48, *p* = 0.02, *BF*_10_ = 2.53, Pz: *t*_(38)_ = 2.16, *p* = 0.04, *BF*_10_ = 1.37), as is shown in Table 4). In the theta band, the oscillatory power for mind-wandering (*M* = 0.12, *SD* = 0.23) was not found to be significantly different from on-task trials (*M* = 0.09, *SD* = 0.20) in the theta band at the frontal sites (Fz: *t*_(38)_ = 0.37, *p* = 0. 71, *BF*_10_ = 0.18). Figure 8 shows there was also no significant difference between mind-wandering and on-task trials in the amplitude of N1, P1, and P3 [P1 component on P7 electrode: mind-wandering (*M* = 4.86, *SD* = 2.35) and on-task (*M* = 4.88, *SD* =2.31), *t*_(38)_ = 0.08, *p* = 0.94, *BF*_10_ = 0.17; N1 component on P7 electrode: mind-wandering (*M* = −6.10, *SD* = 2.78) and on-task (*M* = −6.18, *SD* = 2.54), *t*_(38)_ = 0.34, *p* = 0.74, BF = 0.18 and P8 electrode: mind-wandering (*M* = −4.97, *SD* = 3.05) and on-task (*M* = -4.87, *SD* = 2.95), *t*_(38)_ = 0.47, *p* = 0.64, *BF* = 0.19; and P3 component on Fz electrode: mind-wandering (*M* = 1.80, *SD* = 1.80) and on-task (*M* = 2.05, *SD* = 2.19), *t*_(38)_ = 1.02, *p* = 0.31, *BF*_10_ = 0.28 and Pz electrode: mind-wandering (*M* = 6.24, *SD* = 2.75) and on-task (*M* = 6.22, *SD* = 2.96), *t*_(38)_ = 0.12, *p* = 0.90, *BF*_10_ = 0.17)]. It is too uncertain to tell the difference between mind-wandering and on-task on P8 electrode: mind-wandering (*M* = 5.76, *SD* = 2.94) and on-task (*M* = 6.10, *SD* = 2.69), *t*_(38)_ = 1.50, *p* = 0.15, *BF*_10_ = 0.47.

**Figure 9:**
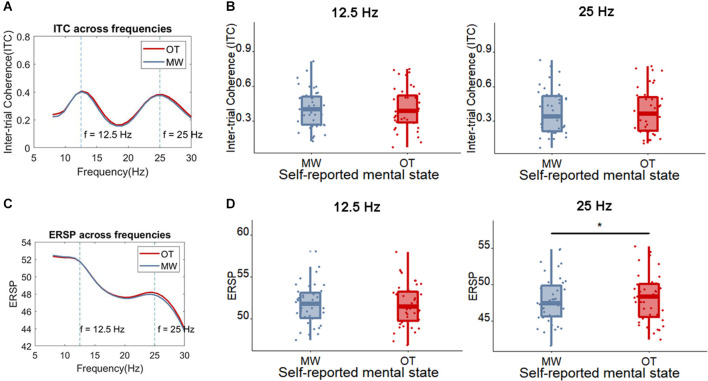
**(A)** The average ITC across frequencies comparing the trials in which participants reported being on-task (OT) vs. mind-wandering (MW). **(B)** Boxplot of the ITC at the frequency of 12.5 Hz and 25 Hz for OT and MW trials, each dot represents one participant. **(C)** The average ERSP ((log)dB) across frequencies for on-task (OT) and mind-wandering (MW) trials. **(D)** Boxplot of the ERSP at the frequency of 12.5 Hz and 25 Hz for OT and MW trials, each dot represents one participant. The only observable difference (indicated with *) is found between on-task and mind-wandering in the ERSP at 25 Hz, which is significantly higher for on-task.

Yet, the ITC of these frequencies did not differ between trials in which participants reported to be focused on the task compared to trials in which they were mind-wandering [12.5 Hz: mind-wandering (*M* = 0.40, *SD* = 0.17), on-task (*M* = 0.41, *SD* = 0.18), *t*_(38)_ = 0.27, *p* = 0.79, *BF*_10_ = 0.18; 25 Hz: mind-wandering (*M* = 0.38, *SD* = 0.2), on-task (*M* = 0.39, *SD* = 0.2), *t*_(38)_ = 0.37, *p* = 0.71, *BF*_10_ = 0.18; see Figure 9 for the ITC and ERSP of on-task and mind-wandering trials].

Interestingly, the mind-wandering trials showed a somewhat weaker ERSP than on-task trials around the harmonic frequency [25 Hz; mind-wandering (*M* = 47.88, *SD* = 3.22), on-task (*M* = 48.13, *SD* = 3.27), *t*_(38)_ = 2.06, *p* = 0.046, *BF*_10_ = 1.16]. No such difference between mind-wandering and on-task was found for the ERSP at the flicker frequency [12.5 Hz; mind-wandering (*M* = 51.70, *SD* = 2.39), on-task (*M* = 51.69, *SD* = 2.55), *t*_(38)_ = 0.068, *p* = 0.95, *BF*_10_ = 0.17].

**Figure 10:**
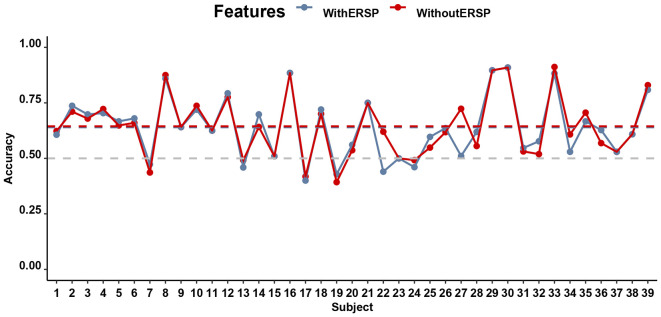
Performance (Accuracy) of the SVM in the SART for all participants: prediction of whether a participant is mind-wandering or on task on the basis of various EEG features. The gray horizontal dashed line indicates the chance level of 50% accuracy while the blue and red dashed lines indicate the mean classifier accuracy with and without the ITC, respectively.

As before, even though there are limited differences between mind-wandering and on-task manifest in both brain and behavior, we still examined whether the signal is usable by a classifier, which may be able to combine multiple sub-threshold signals to make reliable predictions about behavior. Figure 10 shows the accuracy of the mind-wandering classifiers with and without the SSVEP signal, separately for every participant (except one participant who reported no mind-wandering trials). Overall, the classifier is able to predict mind-wandering with an average accuracy of 64.44% (ranging from 39.29% to 91.18% across participants) when the SSVEP signal is in the form of the ERSP at the frequency of 12.5 Hz and 25 Hz is not included. The accuracy was significantly higher (*p* < 0.01) than the corrected chance level (58.68%) considering the sample size (167 on average, ranging from 82 to 217 across participants) and the number of classes (2). When ERSP is included in the classifier, the accuracies range from 40.00% to 90.91% (average accuracy is 63.99%). Yet, there is no significant difference in prediction accuracy between the classifiers that did and did not include ERSP (*t*_(38)_ = 0.52, *p* = 0.61, *BF*_10_ = 0.20).

## Discussion

In this study, we examined whether the SSVEP triggered by flickering stimuli of a SART could help to determine the extent to which an individual is distracted with sticky or more generally task-unrelated spontaneous thoughts. Consistent with existing studies, we found reduced behavioral performance when the spontaneous thoughts were found difficult to disengage from (“sticky”) compared to when it was easy to let go. There was weak evidence for the SSVEP evoked by flickering being reduced in amplitude during more sticky spontaneous thought. Moreover a machine learning classifier achieved a decent accuracy at predicting the stickiness of thoughts on the basis of EEG features including single-trial P1, N1, and P3 alpha and theta oscillations and the ERSP at the SSVEP frequency although the SSVEP did not improve the classification of more vs. less sticky spontaneous thoughts (without the SSVEP: 72.60% and with the SSVEP: 73.35%).

Our observation of differences in SSVEP and behavior with sticky thought is interesting because this stickiness of thoughts may be related to depressive rumination which is a maladaptive form of thinking (Ottaviani et al., 2013, 2015; Marchetti et al., 2016). As such, the stickiness-related SSVEP signal may have interesting applications in future studies of depressive thinking. More specifically, these findings may be usable in clinical settings in the future. For example, if indeed sticky thinking is a light form of rumination, then the detection of sticky thinking may be helpful to catch a depressive relapse at an early stage. In other words, this biomarker—if it is verified in future studies of depressive patients—could potentially contribute to the diagnosis and an early intervention for depression.

In contrast to previous studies, we did not find a difference in behavioral performance between mind-wandering and on-task trials. In addition, we failed to find differences in ERPs between mind-wandering and on-task performance. However, we did find slightly increased alpha-band EEG oscillations while a participant was mind-wandering, which also in previous studies have been shown to be the most consistent indicator of mind wandering (Jann et al., 2010; Mo et al., 2013; Jin et al., 2019; Arnau et al., 2020). We failed to find a difference in the SSVEP evoked by flickering words between subjectively-rated mind-wandering and on-task states. The SSVEP also did not improve the classification accuracy of a machine learning classifier set up to distinguish mind-wandering from being on-task on single trials. It is possible that our failure to find an effect of SSVEP is due to limitations in signal processing. In a separate analysis we used a more state-of-art spatial filtering technique, namely canonical correlation-based spatial filtering to extract SSVEP features from five occipital electrodes (PO3, O1, Oz, O2, and PO4 electrode; Chen et al., 2014). This method removes noise by finding a transformation that maximizes the correlation between the original EEG signal and the evoked potential (which is presumably a signal with less noise, since it has been averaged out). Despite this noise reduction, our conclusion remained the same: the accuracy for predicting stickiness reached 74.16% while the accuracy for predicting mind-wandering reached 65.24%. The SSVEP did not contribute to the prediction of either stickiness or mind-wandering.

Since the SSVEP is a well-known indicator of where on the screen attention is directed (Müller et al., 1998a; Gulbinaite et al., 2019), one may wonder how it could be the case that it fails to detect the attentional disengagement associated with mind-wandering. One possible reason is that in the current study, attention need not be localized to a particular spatial location, but instead just generally at the center of the screen. In other words, the SART can be completed without detailed visual processing. It could therefore be the case that attention was partly directed inwardly to spontaneous thoughts while some global level of attention on bottom-up visual stimuli remained. Specifically, in the task we are studying, there are at least three different possible foci of attention: (a) the visual flickering without processing the letters; (b) the actual letter cases and; (c) endogenous thoughts. It is therefore possible that participants were paying some rudimentary attention to the flickering stimulus without actually processing the letters—leading to a relatively low SSVEP signal that would not change when a participant disengages their attention further during mind-wandering. This is supported by the attenuated SSVEP attend-vs.-unattended effect during covert attention compared to overt attention, i.e., when the eyes are focussed to the center of the screen instead of directly gazing at the stimulus, the difference in SSVEP amplitude between attended and unattended stimulus was significantly lower (Walter et al., 2012).

Another possible explanation for our failure to find SSVEP being reduced during mind-wandering may come from the nature of the task: as the word stimuli kept flickering all the time, participants’ attention was constantly directed to the flickering words. The salience of the flickers may have reduced the occurrence of mind-wandering and made it more difficult to decouple attention from the stimuli. Indeed, the average rate of mind-wandering in the current task is 37.60%, which is lower than in previous studies, which reported rates of 42% and 44% given exactly the same content of thought probes (McVay and Kane, 2013; Jin et al., 2019). This relatively low rate of mind-wandering occurred despite our efforts to increase mind-wandering by priming concerns with a writing task at the start of the experiment and including concern-related works in the task itself (McVay and Kane, 2013). Unlike the degree of the stickiness of spontaneous thoughts, the degree of being on-task was not associated with differences in behavior. Compared to previous studies using the same SART paradigm without flickering stimulus, we could speculate that the flickering might be the major factor why we missed the difference in behavioral performance between mind-wandering and on-task.

Although we started our study with the intention to improve the prediction of spontaneous thought by using the SSVEP, in fact, we found continued challenges in predicting spontaneous thought on a single-trial level. The machine learning classifiers yielded only a moderate level of accuracy, and it is worth noting that the sensitivity of the classifiers is still low (see Table 2), which means the models are biased when classifying the two classes. This bias occurred despite the fact that similar to the study of Jin et al. (2019), we used a practice of oversampling to balance the size of the two classes in the training dataset. The bias in the classifier could be due to the imprecision of the labels based on self-reported thought probes—in other words, participants may not be very good at estimating their own mental states (Weinstein, 2017). This would result in high levels of label noise, which makes it more challenging for the classifier to learn the proper mappings between EEG signal and mind-wandering judgments, and increases the chances of biases. As such, our results should be treated with caution and replicated in larger samples. Moreover, it may be worth the effort to improve label quality by training participants on reporting their own thoughts, as is done for example in studies using microphenomenology (Petitmengin et al., 2019) or Descriptive Experience Sampling (Hurlburt and Akhter, 2006). Another intriguing direction for future research is to complement the subjective labels with more advanced data features. For example, Zanesco (2020) has developed methods to quantify the dynamics of behavior based on autoregressive methods. It could be interesting to examine whether such fluctuations in behavior are related to subjective ratings such as stickiness (see also Irrmischer et al., 2018 for similar ideas).

In this study, we had to dichotomize the continuous rating scales to have sufficient statistical power for comparisons. While we assumed trials within each category to be homogeneous, it remains possible that this dichotomization has affected the results. Future research may explain more continuous classification methods. Another limitation is that the statistics were not corrected for the number of comparisons in this study although multiple comparisons including the ERPs, alpha oscillations, and SSVEP were made. Moreover, the effect of ITC between less sticky trials and more sticky trials did not survive after correction using a false discovery rate. The robustness of this effect needs further consolidation by future studies on sticky thinking.

In summary, we examined whether the SSVEP could track subjective judgments of spontaneous thought including mind-wandering and sticky thought. We found the SSVEP was able to track another dimension of spontaneous thought, namely the difficulty of disengaging from this thought. Moreover, the classification of the stickiness of thoughts with EEG features achieved a moderate accuracy. Surprisingly, it did not distinguish between mind-wandering and being on task. Future studies should elucidate the boundary conditions under which the SSVEP operates—for example, what dimensions of thought it is most sensitive to and in what paradigms.

## Data Availability Statement

The raw data supporting the conclusions of this article will be made available by the authors, without undue reservation.

## Ethics Statement

The studies involving human participants were reviewed and approved by Research Ethics Review Committee, University of Groningen. The patients/participants provided their written informed consent to participate in this study.

## Author Contributions

HY: funding acquisition, data curation, formal analysis, investigation, methodology, project administration, resources, software, validation, visualization, writing—original draft, writing—review and editing. KP: conceptualization, methodology, writing—review and editing. MV: conceptualization, funding acquisition, methodology, project administration, resources, supervision, validation, writing—review and editing.

## Funding

This work was supported by the China Scholarship Council (CSC), with the grant (201808330419) awarded to HY.

## Footnotes

^1^We tried to maintain the flickering also during the ISI, but this majorly bothered the participants, and for this reason we decided to abolish it.

## Appendix: Writing Instruction

Before starting the SART experiment, participants were given a short writing assignment, using the instructions below. The objective of this assignment was to trigger self-related and ruminative thinking.

You will now have ten minutes to write about the event that you just nominated. In your writing, I’d like you to really let go and explore your deepest emotions and thoughts about this specific issue. You may write about more than one aspect of the event. Some people find it best to explore their emotions and then try to come to an understanding of the event(s). Do your best to try to “tie it all together” at the end of your writing. We ask that you do not include any information that could identify you as we want this writing to remain confidential and for you to feel free to be able to write things you would not want tied to you. Only the researchers involved in this project will have access to read your essays. Therefore, your confidentiality is assured and your name will never be linked to anything you write. The important thing is that you really let go and dig down to your very deepest emotions and thoughts about the negative event and explore them in your writing. You have ten minutes to write about the nominated event. Following the ten-minute writing task, the EEG experiment began.
