# Supplementary material for: The steady state visual evoked potential (SSVEP) tracks “sticky” thinking, but not more general mind-wandering
Source: Front Hum Neurosci. 2022 Aug 11;16:892863. doi: 10.3389/fnhum.2022.892863 (PMC9402933; doi:10.3389/fnhum.2022.892863)
Supplement: Supplementary file 1 [file Table_1.DOCX]

# Supplementary material

Table A1. Proportions of self-reported mind-wandering and on-task, less sticky and more sticky thoughts for each participant (“other” represents an answer which was neither mind-wandering nor on-task in terms of task-relatedness of thought, and an answer which was neither less sticky nor more sticky in terms of stickiness of thought)

| Participant No. | Task-relatedness of thought | | | Stickiness of thought | | |
| --- | --- | --- | --- | --- | --- | --- |
|  | Mind-wandering | Other | On-task | Less sticky | Other | More sticky |
| 1 | 0.27 | 0.06 | 0.67 | 0.49 | 0.15 | 0.36 |
| 2 | 0.15 | 0.35 | 0.5 | 0 | 0.61 | 0.39 |
| 3 | 0 | 0.44 | 0.56 | 0.85 | 0.15 | 0 |
| 4 | 0.25 | 0.16 | 0.58 | 0.96 | 0.04 | 0 |
| 5 | 0.63 | 0.14 | 0.22 | 0 | 0.04 | 0.96 |
| 6 | 0.31 | 0.02 | 0.67 | 0 | 0 | 1 |
| 7 | 0.5 | 0.27 | 0.23 | 0.78 | 0.17 | 0.06 |
| 8 | 0.48 | 0.13 | 0.39 | 0.04 | 0.66 | 0.3 |
| 9 | 0.13 | 0.08 | 0.79 | 1 | 0 | 0 |
| 10 | 0.34 | 0.04 | 0.62 | 0.14 | 0.06 | 0.8 |
| 11 | 0.23 | 0.17 | 0.6 | 0.83 | 0.08 | 0.08 |
| 12 | 0.36 | 0.46 | 0.19 | 0.35 | 0.23 | 0.42 |
| 13 | 0.19 | 0.12 | 0.69 | 0.17 | 0.08 | 0.75 |
| 14 | 0.46 | 0.04 | 0.5 | 0.45 | 0.23 | 0.32 |
| 15 | 0.29 | 0.21 | 0.5 | 0.54 | 0.02 | 0.44 |
| 16 | 0.46 | 0.18 | 0.36 | 0.15 | 0.23 | 0.62 |
| 17 | 0.55 | 0.37 | 0.08 | 0.08 | 0.15 | 0.77 |
| 18 | 0.36 | 0.19 | 0.45 | 0.39 | 0.21 | 0.4 |
| 19 | 0.21 | 0.17 | 0.62 | 0.23 | 0.25 | 0.52 |
| 20 | 0.46 | 0.17 | 0.37 | 0.57 | 0.19 | 0.25 |
| 21 | 0.3 | 0.33 | 0.38 | 0.32 | 0.08 | 0.6 |
| 22 | 0.23 | 0.34 | 0.43 | 0.5 | 0.25 | 0.25 |
| 23 | 0.46 | 0.2 | 0.33 | 0.44 | 0.21 | 0.36 |
| 24 | 0.44 | 0.19 | 0.37 | 0.06 | 0.49 | 0.44 |
| 25 | 0.53 | 0.06 | 0.41 | 1 | 0 | 0 |
| 26 | 0.44 | 0.04 | 0.52 | 0 | 0.13 | 0.87 |
| 27 | 0.36 | 0.08 | 0.56 | 0.02 | 0.17 | 0.81 |
| 28 | 0.57 | 0.23 | 0.2 | 0.19 | 0.15 | 0.66 |
| 29 | 0.38 | 0.06 | 0.56 | 0.55 | 0.33 | 0.12 |
| 30 | 0.59 | 0.37 | 0.04 | 0.08 | 0.35 | 0.56 |
| 31 | 0.88 | 0.04 | 0.08 | 0.37 | 0.36 | 0.27 |
| 32 | 0.42 | 0.06 | 0.52 | 0.36 | 0.33 | 0.31 |
| 33 | 0.48 | 0.19 | 0.33 | 0.11 | 0.04 | 0.85 |
| 34 | 0.54 | 0.4 | 0.06 | 0.98 | 0.02 | 0 |
| 35 | 0.27 | 0.27 | 0.46 | 0.1 | 0.35 | 0.55 |
| 36 | 0.21 | 0.21 | 0.58 | 0.25 | 0.17 | 0.59 |
| 37 | 0.53 | 0.27 | 0.21 | 0.83 | 0.08 | 0.08 |
| 38 | 0.34 | 0.44 | 0.22 | 0.04 | 0.31 | 0.64 |
| 39 | 0.44 | 0.25 | 0.31 | 0.14 | 0.27 | 0.58 |
| 40 | 0.13 | 0.31 | 0.56 | 0.27 | 0.19 | 0.54 |
